# A manually denoised audio-visual movie watching fMRI dataset for the studyforrest project

**DOI:** 10.1038/s41597-019-0303-3

**Published:** 2019-11-29

**Authors:** Xingyu Liu, Zonglei Zhen, Anmin Yang, Haohao Bai, Jia Liu

**Affiliations:** 1 Beijing Key Laboratory of Applied Experimental Psychology, Beijing Normal University, Beijing, 100875 China; 2 Faculty of Psychology, Beijing Normal University, Beijing, 100875 China

**Keywords:** Sensory processing, Perception, Visual system, Language, Functional magnetic resonance imaging

## Abstract

The data presented here are related to the studyforrest project that uses the movie ‘Forrest Gump’ to map brain functions in a real-life context using functional magnetic resonance imaging (fMRI). However, neural-related fMRI signals are often small and confounded by various noise sources (i.e., artifacts) that makes searching for the signals induced by specific cognitive processes significantly challenging. To make neural-related signals stand out from the noise, the audio-visual movie watching fMRI dataset from the project was denoised by a combination of spatial independent component analysis and manual identification of signals or noise. Here, both the denoised data and the labeled decomposed components are shared to facilitate further study. Compared with the original data, the denoised data showed a substantial improvement in the temporal signal-to-noise ratio and provided a higher sensitivity in subsequent analyses such as in an inter-subject correlation analysis.

## Abstract (summary)

**Table:** 

Measurement(s)	Blood Oxygen Level-Dependent Functional MRI
Technology Type(s)	data transformation
Sample Characteristic - Organism	Homo sapiens

Machine-accessible metadata file describing the reported data: 10.6084/m9.figshare.10266554

## Background & Summary

In daily life, we are constantly processing a vast amount of information that dynamically and rapidly flows into our minds through multiple sensory channels. One of the ultimate goals of cognitive neuroscience is to understand how stimuli encountered in the dynamic natural environment are processed by neural circuits. However, most cognitive neuroscience studies are limited to simple stimuli and dull conditions^1^. Recently, researchers have begun to examine how the brain works in response to the dynamic complexity of natural conditions, using vivid movies as stimuli, and taking advantage of the fact that movies can reflect a wealth of real-life content, thus triggering naturally occurring brain states and dynamics^2–6^. To facilitate the study of brain functions in complex life environments, the studyforrest project has collected and shared a set of blood oxygenation level dependent (BOLD) functional magnetic resonance imaging (fMRI) data from participants who were watching the two-hour movie ‘Forrest Gump’ (R. Zemeckis, Paramount Pictures, 1994). A comprehensive set of auxiliary data have also been acquired^7^. Detailed information on the studyforrest project can be found in the related data papers^8,9^ and the associated website (http://www.studyforrest.org). The studyforrest dataset provides a versatile resource for studying information processing under real-life conditions.

Due to its unprecedented capacity to capture whole-brain neural response patterns with high spatial and temporal resolution, fMRI has become the standard workhorse technique for investigating human brain function. However, fMRI data is very noisy. The BOLD responses induced by neural activity are often very small and are comprised of various noise sources (i.e., artifacts). Artifacts can be induced by hardware instabilities (e.g., spiking), head motion, or a multitude of physiological fluctuations of non-neural origin, including cardiac and respiratory noise^10^. Head motion induces motion-by-susceptibility interactions and leads to significant confounding signal variance^11–14^. Physiological artifacts occur at a relatively high frequency (~1 Hz and ~0.3 Hz for cardiac and respiratory cycles^15,16^, respectively); nevertheless, they can be aliased into lower frequencies in which neural-related signals reside for the standard repetition time (TR; ~2 s)^17^. If not carefully cleaned up, such confounding artifacts may result in biases or errors in fMRI results and the interpretation thereof^18^. Therefore, it is highly desirable to remove those artifacts to obtain reliable and accurate measures of brain activity (in terms of response magnitude and functional connectivity), particularly for a public dataset such as the studyforrest dataset, that could be used to answer a number of broad cognitive neuroscience questions. To this end, the audio-visual movie watching fMRI data from the studyforrest project were denoised in this study by combining spatial independent component analysis (ICA) with the manual identification of signals and artifacts; both the denoised data and the labeled decomposed components are presented so they can be used for further study.

Spatial ICA is a proven, powerful tool for blind source fMRI data separation^19,20^. It attempts to decompose the fMRI data into a set of statistically independent components (ICs): spatial maps and associated time series. Extensive empirical studies have demonstrated that each of the ICs generally represents either a neural-related signal or a certain type of artifact. As a result, different types of artifact components can be identified from a set of ICs automatically generated by ICA that can then be filtered out from the original data, successfully achieving ICA-based artifact removal^21^. However, sorting the artifact components is challenging since the relative contributions of each type of artifact vary widely across different scanners, subjects, and acquisition runs. In addition, some artifacts share similar spatial, temporal and/or spectral characteristics as the signal of interest. So far, visual inspection and manual selection remains the gold standard for component classification^19,21,22^. Even for automatic classification techniques, manual classification of some ICs is still required to train a model^21^. To clean the data as completely and accurately as possible, an ICA-based manual classification method was adopted in this study. In other words, to denoise the data, a spatial ICA was performed on the original fMRI data of each run from each participant^23,24^. All components from the ICA were then manually sorted into signals and different artifact sources. The denoised data were finally generated by removing the components classified as artifacts from the original data.

## Methods

### Source data

An audio-visual movie watching fMRI dataset from the studyforrest project was collected from participants watching the movie ‘Forrest Gump’. Fifteen participants watched the two-hour audio-visual movie ‘Forrest Gump’ while undergoing fMRI scanning with a 3 Tesla Philips Achieva dStream MRI scanner. Ethical approval was obtained from the Ethics Committee of the Otto-von-Guericke University and all participants gave informed consent before participation. The acquisition parameters for the studyforrest audio-visual movie watching fMRI dataset were previously provided by Hanke *et al*.^8,9^. In summary, the data were acquired with the aforementioned whole-body 3 T scanner equipped with a 32-channel head coil using T2*-weighted gradient-echo echo-planar sequences (TR = 2 s, echo time [TE] = 30 ms, flip angle = 90°, SENSE factor = 2, voxel size = 3 × 3 × 3 mm). The approximately 2-hour long slightly re-edited movie was split into 8 segments (average length ≈ 15 minutes) that were presented in chronological order in 8 runs. The auxiliary structural MRI data scans were recorded in the same MRI scanner using a three-dimensional turbo field echo sequence (TR = 2500 ms, TI = 900 ms, flip angle = 8 degrees, TE = 5.7 ms, voxel size = 0.67 × 0.67 × 0.67 mm).

### Data denoising procedures

**Fig. 1:**
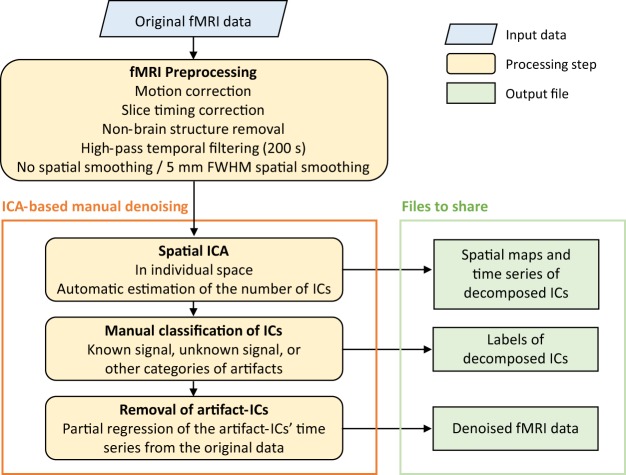
A schematic overview of the four-step denoising workflow. Preprocessing was performed on the original fMRI data and included motion correction, slice timing correction, non-brain structure removal, high-pass filtering (200 s cut-off); all steps were performed without or with spatial smoothing (i.e., FWHM = 0 mm or FWHM = 5 mm). A spatial ICA was next run on the preprocessed data in individual space, producing spatial maps and time series files of the decomposed ICs for each run and each participant. All decomposed ICs were then manually classified into either a known signal, unknown signal, or different categories of artifacts. Finally, all the ICs classified as artifacts were filtered out, producing the final denoised fMRI data. (fMRI: functional magnetic resonance imaging, FWHM: full width half maximum, IC: independent component, ICA: independent component analysis).

Since the contributions of each type of artifact vary significantly across participants and acquisition runs, the studyforrest audio-visual movie fMRI dataset was denoised separately for each run from each participant (i.e., 8 × 15 = 120 runs, in total) following a four-step procedure that included preprocessing, ICA decomposition, manual classification of ICs, and artifact removal (Fig. 1).

#### Preprocessing

Preprocessing of the functional images was performed using FEAT (FMRIB Expert Analysis Tool version 6.00, part of FMRIB’s Software Library [FSL; www.fmrib.ox.ac.uk/fsl]), and included motion correction, slice timing correction, brain extraction and high-pass temporal filtering (200 s cut-off)^25^. Notably, spatial smoothing can increase the signal-to-noise ratio, but can compromise fine-grained spatial information in some cases; thus, the denoising procedure was performed on both unsmoothed and smoothed (Gaussian kernel, FWHM = 5 mm) preprocessed data to maximize the quality of the data accordingly.

#### Spatial ICA

A spatial ICA was performed on each run from each participant in individual space using a probabilistic ICA algorithm implemented with MELODIC (version 3.15) from the FSL^23,24^ using default parameters. MELODIC decomposes the four-dimensional functional data into a set of spatial independent maps, each with their own associated time series. Briefly, each spatial map characterizes the spatial distribution of a specific source (neural-related signal or artifacts), and the time series encodes how that spatial map contributes to the data over time. The number of components is automatically calculated using a Bayesian dimensionality estimation technique^26^.

#### Manual classification of ICs

To maximize IC classification accuracy, two raters with expert knowledge of neuroanatomy (i.e., X.L. and Z.Z) worked together to reach an agreement regarding each IC label using melview (https://git.fmrib.ox.ac.uk/fsl/melview). Specifically, the ICs were sorted into neural-related signals or artifacts according to three complementary pieces of information: the IC spatial map, its time series, and its power spectral density (i.e., the magnitude of the Fourier transform of the time series). Seven categories of artifact-related ICs (A-ICs) were defined: hardware (MRI related noise [mostly susceptibility]), participants’ head motion, physiology (arteries, cerebrospinal fluid [CSF], veins [mostly sagittal sinus], and white matter [including deep veins of the brain]) and unclear sources (unclassified noise). Two categories of signal-ICs (S-ICs) were defined. One was defined as known signal, reflecting well-known characteristics of neural-related signals. The other category of S-ICs was defined as unknown signal, comprising neither typical characteristics of neural-related signals nor clear characteristics of artifacts. The characteristics of different IC categories are summarized in Table 1; examples of labeled S-ICs and A-ICs are provided in Supplementary Figs. 1–9.

**Table 1:** Spatial, temporal and power characteristics of different categories of ICs. (CSF: cerebral spinal fluid, MRI: magnetic resonance imaging, S-IC: signal-independent component, A-IC: artifact-related independent component).

Category	Spatial map	Time series	Power spectrum
Known signal	Clear clusters in gray matter or subcortical structures; usually bilateral	No sudden jumps or gradual drifts	Predominantly in low frequencies (<0.1 Hz)
Head motion	Ring-like shape or stripes at the intensity edges of the brain	Sudden jumps and/or gradual drifts; correlated well with head motion parameters from motion correction	Broad bands, could reside in both low- and high-frequencies
Arteries	Tightly coupled with known anatomical structures	Regular oscillatory patterns; no sudden jumps or gradual drifts	Generally dominated by high frequencies (>0.1 Hz); however, could be aliased into low frequencies (<0.1 Hz)
CSF			
White matter			
Veins		No sudden jumps or gradual drifts	Predominantly in low frequencies (<0.1 Hz)
MRI related	Abrupt intensity changes in slice direction or stripes along the phase encoding direction	Sudden jumps and/or sudden changes of oscillation patterns	Predominantly in high frequencies (>0.1 Hz)
Unclassified noise	Large number of small clusters over the brain; not clearly reflective of any known structures	Sudden jumps and/or sudden changes of oscillation patterns	Predominantly in high frequencies (>0.1 Hz) or very low frequencies (<0.01 Hz)
Unknown signal	Usually a mixture of signals and noise, both criteria of S-IC and A-IC are met or partially met		

To evaluate the inter-rater classification reliability, a third rater (i.e., A.Y.) independently labeled ICs from a randomly chosen run from each participant. The percentage of ICs whose labels the third rater and the original raters agreed upon was then used to measure the inter-rater classification reliability. The percentage of agreed labeled ICs was 76.81% (standard error of the mean [SEM] = 0.60%) for the smoothed data and 73.29% (SEM = 1.17%) for the unsmoothed data in the nine-catogory classification task. Since the most important thing in denoising the data is to distinguish between S-ICs (not to be removed) and A-ICs (to be removed), the inter-rater agreement was further evaluated with regard to this binary classification; agreement reached 90.83% (SEM = 0.83%) for the smoothed data and 91.20% (SEM = 1.50%) for the unsmoothed data. Taken together, these results indicated that this denoising approach has good reliability.

The fact that signal and noise may be mixed in one single IC because their spatial variations are not independent presents a challenge in IC classification. Since the priority in cleaning the fMRI data was to reduce noise while preserving as much of the signal of interest as possible^21^, preference was given to these mixed ICs and they were labeled as unknown signals (i.e., S-ICs) in case they could not be confidently classified as A-ICs. Therefore, it is worth noting that an IC may have been inadequately labeled as a signal in the present procedure, and vice versa.

#### Removal of A-ICs

After identifying the S-ICs and A-ICs, two possible strategies can be used to clean the data. The first is to reconstruct the data from the S-ICs (combining spatial maps with their associated time series and calculating a total)^27^; the second is to clean the data by partial regression of the time series of the A-ICs from the original data^28^. Here, the second strategy was used because it would preserve the stochastic variation inherent to the denoised data, thus allowing for possible ‘null hypothesis’ testing in subsequent data analyses (e.g., a general linear model analysis)^29^. If needed, users can easily reconstruct the data by the first strategy using the S-ICs provided.

### Data Records

Following the denoising procedure, two types of data were produced for each run from each participant. The first type of data consisted of the denoised fMRI data. The second type of data consisted of the spatial maps and time series of the decomposed ICs as well as their manually classified labels. All these data and their attached description files are available from the OpenNeuro portal (dataset accession number: ds001769, version 1.2.2) at 10.18112/openneuro.ds001769.v1.2.2^30^. The dataset was formatted to follow Brain Imaging Data Structure (BIDS) specification and the BIDS enhancement proposal BEP003 on Common derivatives specifications^31^. Currently, BEP003 is still not finalized and not supported by bids-validator; therefore, a .bidsignore file declaring ignored derivative data files and a made-up scan (i.e., sub-phantom) was included to make the dataset pass the validation.

### Denoised fMRI data

Location: sub-<participant_id>/ses-movie/func/sub-<participant_id>_ses-movie_task-movie_run-<run_id>_space-T1w_desc-preproc_desc-{sm5,unsm}_desc-denoised_bold.nii.gz

File format: NIfTI, gzip-compressed.

### Spatial maps of decomposed ICs

Location: sub-<participant_id>/ses-movie/func/sub-<participant_id>_ses-movie_task-movie_run-<run_id>_space-T1w_desc-preproc_desc-{sm5,unsm}_desc-MELODIC_components.nii.gz

File format: NIfTI, gzip-compressed.

### Time series of decomposed ICs

Location: sub-<participant_id>/ses-movie/func/sub-<participant_id>_ses-movie_task-movie_run-<run_id>_space-T1w_desc-preproc_desc-{sm5,unsm}_desc-MELODIC_mixing.tsv

File format: text (tab-separated-values)

### Labels of ICs

Location: sub-<participant_id>/ses-movie/func/sub-<participant_id>_ses-movie_task-movie_run-<run_id>_space-T1w_desc-preproc_desc-{sm5,unsm}_desc-MELODIC_componentLabels.txt

File format: text (comma-separated-values). Columns indicate the IC index, classification category, and whether it was removed by the denoising procedure. The last row lists the indices of all removed ICs.

### Technical validation

The technical quality of the datasets was validated in three ways. First, the information on IC labels was summarized and compared to previous studies. Second, the denoising procedure was shown to increase the temporal signal-to-noise ratio (tSNR) of the data. Finally, the denoising procedure was confirmed to selectively increase the inter-subject correlation (ISC) in movie watching-related brain regions, indicating potential benefits to further neuroscience-oriented data analyses.

### Artifacts accounted for more variance than signals

**Fig. 2:**
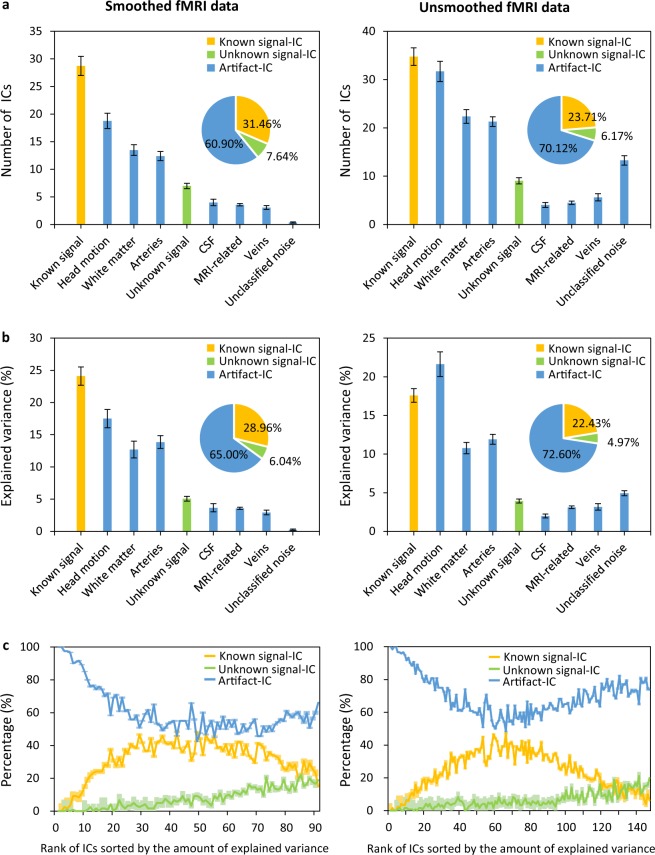
Artifact ICs accounted for a large proportion of ICs and variances in both smoothed (left column) and unsmoothed (right column) fMRI data. **(a)** The number of ICs classified into different categories is shown. The number of ICs was averaged across all runs and participants. **(b)** The explained variance of ICs in different categories is shown. The explained variance of all ICs in each category was totaled at each run, then averaged across all runs and participants. **(c)** The proportions of known signal-ICs, unknown signal-ICs, and artifact-ICs for each rank are shown. ICs from each run were ranked according to the amount of variance they explained, and the proportions of the three superordinate IC categories were summarized across all runs and participants for each rank. The error bars in (**a**) and (**b**) and shaded areas in (**c**) denote the standard error (SEM) of participants (n = 15). (fMRI: functional magnetic resonance imaging, IC: independent component, CSF: cerebral spinal fluid).

As shown in Fig. 2a, there were more A-ICs than S-ICs regardless of whether the data were smoothed during preprocessing. On average, 91 ICs were produced from 1 run for the smoothed data and 147 ICs for the unsmoothed data. Among these ICs, 60.90% and 70.12% ICs were classified as artifacts for the smoothed and unsmoothed data, respectively, consistent with results from previous fMRI denoising studies^29,32^. These ICs explained 65.00% of the smoothed data variance and 72.60% of the unsmoothed data variance, respectively. Among them, physiological factors (arteries, CSF, veins, and white matter) and head motion were two main sources of noise, 33.14% and 17.52% of the smoothed data variance and 27.84% and 21.63% of the unsmoothed data variance, respectively (Fig. 2b). Moreover, the amount of variance explained by individual A-IC was often larger than that of individual S-ICs. When ICs were ordered by the amount of explained variance, on average, more than eight ICs in the top 10 were A-ICs (Fig. 2c). These results indicated that the fMRI data was remarkably noisy, attesting to the importance of data denoising in fMRI data analysis.

In addition, the effects of spatial smoothing in ICA decomposition and the consistency of subsequent manual classifications were examined. First, a mutual maximum similarity criterion was used to identify consistent IC pairs from ICA decompositions of smoothed and unsmoothed data. In other words, if an IC from smoothed data showed maximum similarity with an IC from unsmoothed data in both spatial map and time series, and vice versa, these two ICs were considered to be successfully combined as a consistent IC pair. The similarity of two ICs in their spatial maps and time series was measured using a Pearson correlation coefficient. Since unsmoothed data produced more ICs than the corresponding smoothed data overall, it is not possible for all ICs from unsmoothed data to be successfully paired with a consistent IC from the smoothed data. Therefore, the proportion of successfully paired ICs in the smoothed data was calculated as an overall measure of the consistency of two decompositions for each run. On average, 84.69% of ICs from the smoothed data were successfully paired with a consistent IC from the unsmoothed data. Specifically, 89.85% of known S-ICs, 69.30% of unknown S-ICs and 83.96% of A-ICs from the smoothed data had consistent ICs from the corresponding unsmoothed data. Second, the agreement of the manual classification of the consistent IC pairs was examined. The consistent IC pairs were generally classified into the same category out of nine categories (on average = 82.34%) despite of some mismatches (Please see Supplementary Fig. 10 for details).

### Artifact removal substantially improved the tSNR of the data

**Fig. 3:**
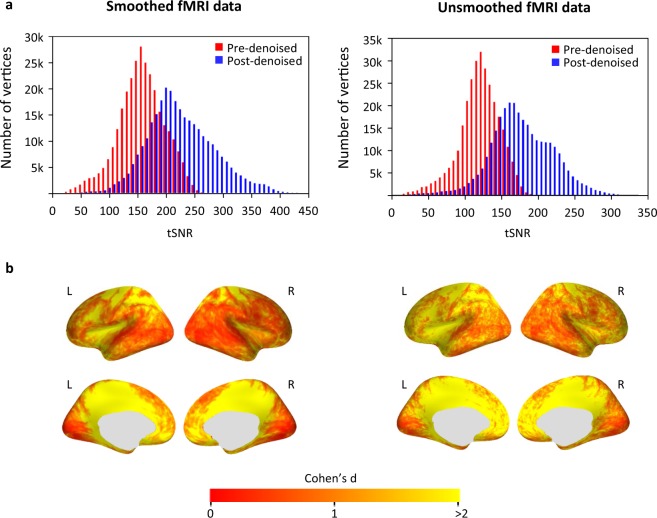
Denoised fMRI data showed higher tSNR than the original data (left: smoothed fMRI data; right: unsmoothed fMRI data). **(a)** Histograms of the tSNR values across all vertices on the fsaverage surface are shown. The tSNR was calculated for each vertex and averaged across all runs and participants. **(b)** The Cohen’s d effect size of the tSNR change is displayed on the fsaverage surface. Cohen’s d was calculated as the mean differences between the tSNR from pre- and post-denoised fMRI data (n = 15 participants), divided by the pooled standard deviation. (fMRI: functional magnetic resonance imaging, tSNR: temporal signal-to-noise ratio, L: left hemisphere, R: right hemisphere).

Here, the tSNR of the data was significantly increased following data denoising. The tSNR was defined as the ratio between the mean of a time series and its standard deviation for each vertex. The fMRI data from each run and each participant were transformed onto the fsaverage surface. The tSNR was then calculated and group-averaged for both the original and denoised data. The denoised data had a substantially higher tSNR than the original data for both smoothed and unsmoothed data (Fig. 3a). The improvement in tSNR in the smoothed data was slightly smaller, possibly because some noise may have already been removed by spatial smoothing. The increase in tSNR occurred across the whole brain, with larger improvements in the cingulate, precuneus, and insular cortex; these are regions where artifacts appeared more (Fig. 3b). Taken together, these results demonstrate the artifact removal process efficacy.

### Artifact removal selectively increased the ISC in task-related brain regions

**Fig. 4:**
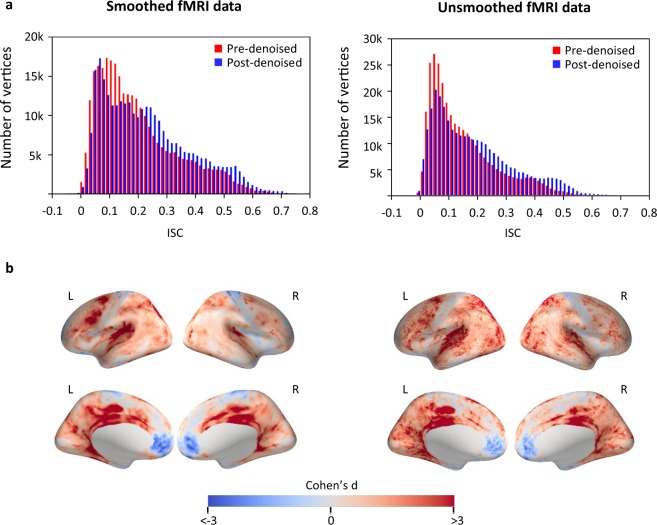
Denoised fMRI data showed higher ISC than the original data in movie watching-related brain regions (left: smoothed fMRI data; right: unsmoothed fMRI data). **(a)** Histograms of ISCs across all vertices on the fsaverage surface are shown. The ISC value was averaged across all runs and participants. **(b)** The Cohen’s d effect size of ISC changes at each vertex on the fsaverage surface is shown. The ISC coefficients were first converted into z-values using a Fisher’s r-to-z transformation. Next, the Cohen’s d value was calculated as the differences in means between the z-values from pre- and post-denoised fMRI data (n = 8 runs), divided by the pooled standard deviation. (fMRI: functional magnetic resonance imaging, ISC: inter-subject correlation, L: left hemisphere, R: right hemisphere).

The removal of artifacts was shown to benefit further research aimed at answering neuroscience questions using ISC analysis as an example. ISC analysis calculated the voxel-wise correlation of BOLD signals between participants. It has been widely used to discover synchronization in brain activity across individuals and localize complex cognitive processes, especially for naturalistic stimulus paradigms such as movie watching^2,4^. The ISC of each per participant and the remaining n-1 participants was calculated after transforming the fMRI data onto the fsaverage surface and a group-averaged ISC map was derived across runs and participants for both original and denoised fMRI data. As shown in Fig. 4a, ISC was comprehensively enhanced in the denoised data, with the average ISC value right-shifted for both smoothed and unsmoothed data. Consistent with previous studies on ISC during movie watching, visual cortices, auditory cortices, precuneus, superior temporal sulcus, and temporal parietal junction showed high ISC in both the pre- and post-denoised data^2,33^. Nonetheless, the primary motor cortex, somatosensory cortex, and medial prefrontal cortex showed very low ISC (Supplementary Fig. 11). Particularly, a clear disassociation of the ISC change produced by the denoising procedure was observed. After denoising, the ISC from the high ISC areas were considerably increased whereas the ISC from the low ISC areas were decreased (Fig. 4b). This disassociation of the denoising effects in different areas indicated that the present denoising procedure specifically enhanced neural-related ISC and weakened ISC from non-neural sources.

## Supplementary information

Supplementary figures

## Data Availability

Preprocessing was performed using FEAT (www.fmrib.ox.ac.uk/fsl). ICA was performed with MELODIC v3.15 (https://fsl.fmrib.ox.ac.uk/fsl/fslwiki/MELODIC) and IC classifications were manually performed using melview (https://git.fmrib.ox.ac.uk/fsl/melview). All code for data denoising and technical validation is available on github.com/xingyu-liu/studyforrest_denoise.
