# Supplementary material for: A manually denoised audio-visual movie watching fMRI dataset for the studyforrest project
Source: Sci Data. 2019 Nov 29;6:295. doi: 10.1038/s41597-019-0303-3 (PMC6884625; doi:10.1038/s41597-019-0303-3)
Supplement: Supplementary file 1 — Supplementary figures [file 41597_2019_303_MOESM1_ESM.pdf]

Supplementary Figures

Supplementary Figure 1 2
Supplementary Figure 2 3
Supplementary Figure 3 4
Supplementary Figure 4 5
Supplementary Figure 5 6
Supplementary Figure 6 7
Supplementary Figure 7 8
Supplementary Figure 8 9
Supplementary Figure 9 10
Supplementary Figure 10 11
Supplementary Figure 11 12

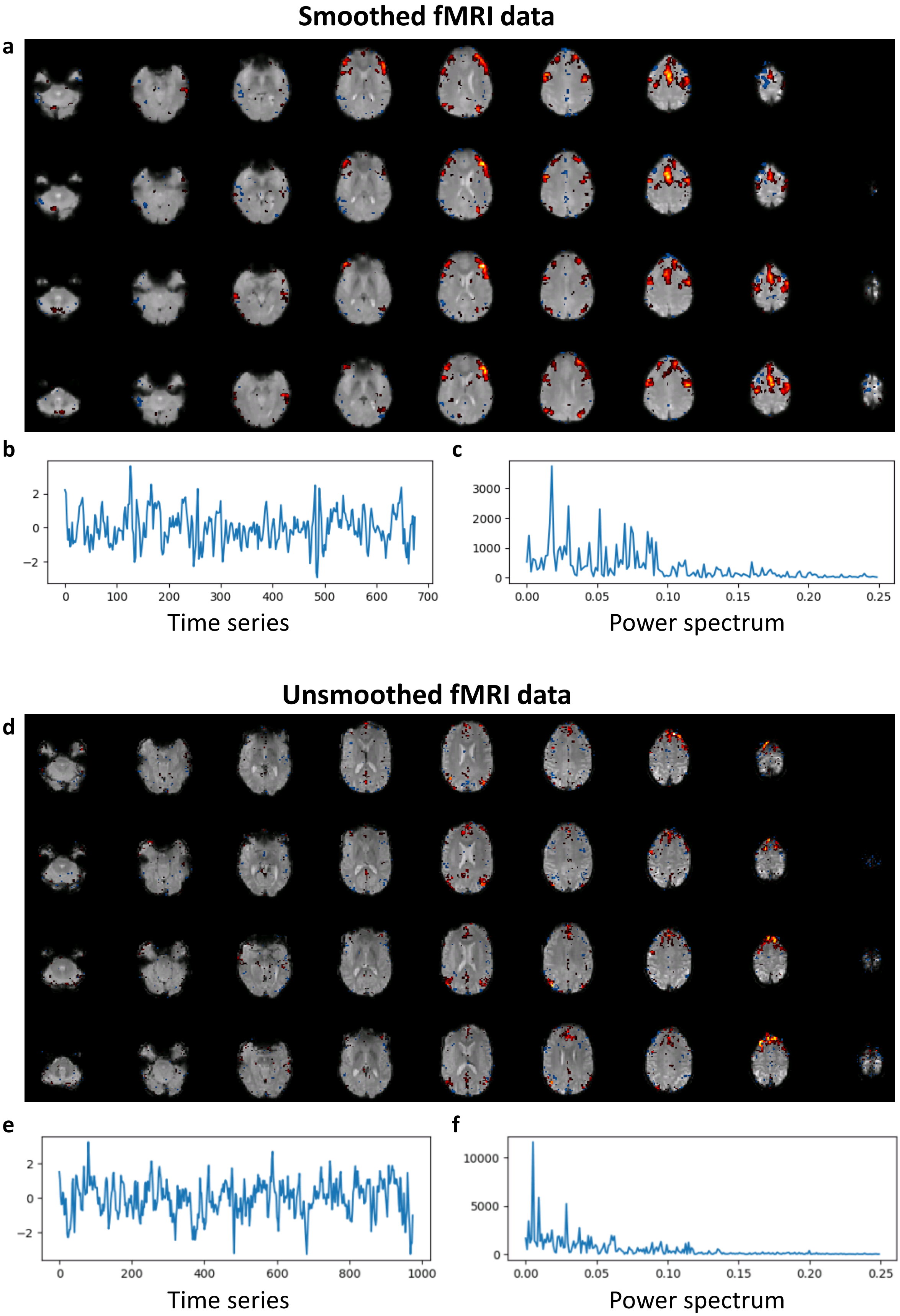

Supplementary Figure 1. Example ICs classified as known signal (top: smoothed fMRI data [sub-20, run-8, IC-034]; bottom: unsmoothed fMRI data [sub-09, run-4, IC-054]). The spatial map of the ICs (thresholded at $Z > 2.3$) showed that strong activities mainly happened within the grey matter **(a, d)**. The time series of the ICs showed a low-frequency oscillation pattern, without sudden jumps or gradual change **(b, e)**. The spectral power of the ICs' time series lied primarily in low-frequency band (i.e., 0.01-0.1Hz) **(c, f)**.

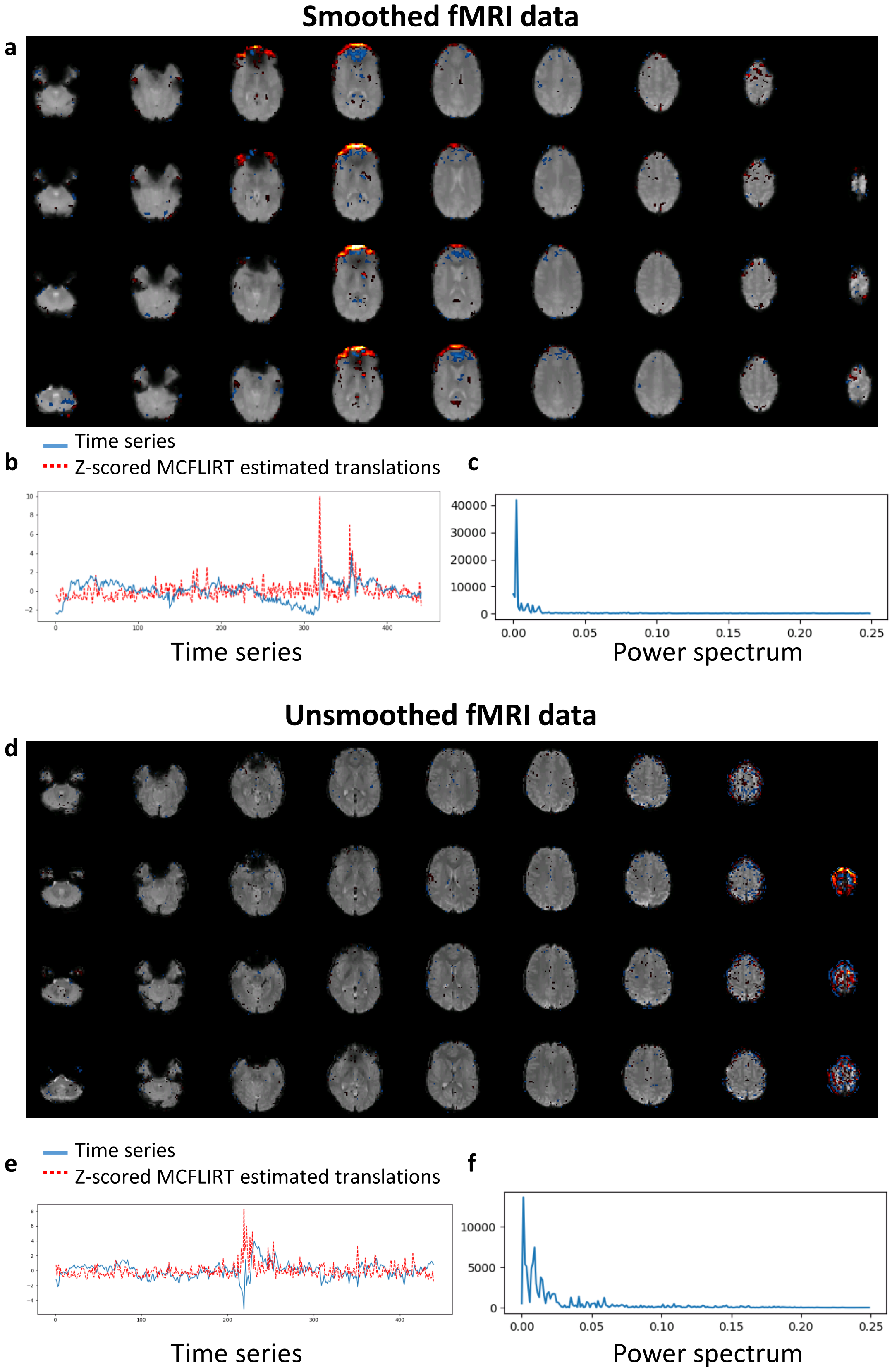

Supplementary Figure 2. Example ICs classified as head motion artifacts (top: smoothed fMRI data [sub-15, run-2, IC-001]; bottom: unsmoothed fMRI data [sub-04, run-6, IC-001]). The spatial map of the ICs (thresholded at $Z > 2.3$) showed that the voxels with large intensities mainly located at brain boundary in a ring shape **(a, d)**. The time series of the ICs contained several sudden jumps which agreed well with one of head motion parameters from motion correction (red dot line) **(b, e)**. The spectral power of the ICs' time series lied primarily in low-frequency band (i.e., 0.01-0.1Hz) **(c, f)**.

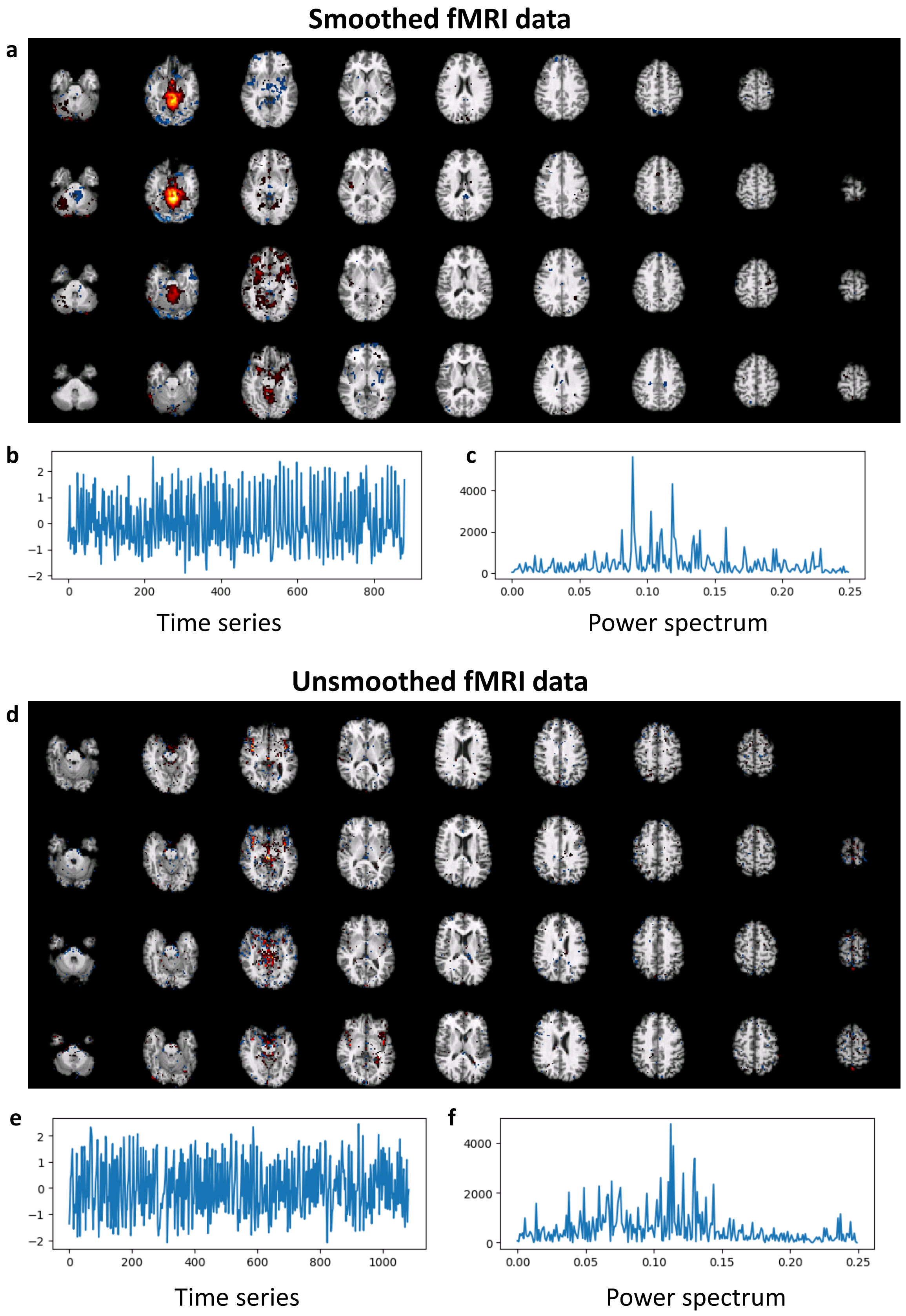

Supplementary Figure 3. Example ICs classified as arteries artifacts (top: smoothed fMRI data [sub-01, run-2, IC-006]; bottom: unsmoothed fMRI data [sub-02, run-7, IC-18]). The spatial map of the ICs (thresholded at Z > 2.3) showed that the voxels with large intensities mainly located in arterial vessels, especially around the cerebral crus and the insular area, which could be observed more clearly with a structural image as underlay **(a, d)**. The time series of the ICs showed a high-frequency oscillation pattern, without sudden jumps or gradual change **(b, e)**. A large portion of the power of the ICs' time series appeared beyond the low-frequency band (i.e., 0.01-0.1Hz) **(c, f)**.

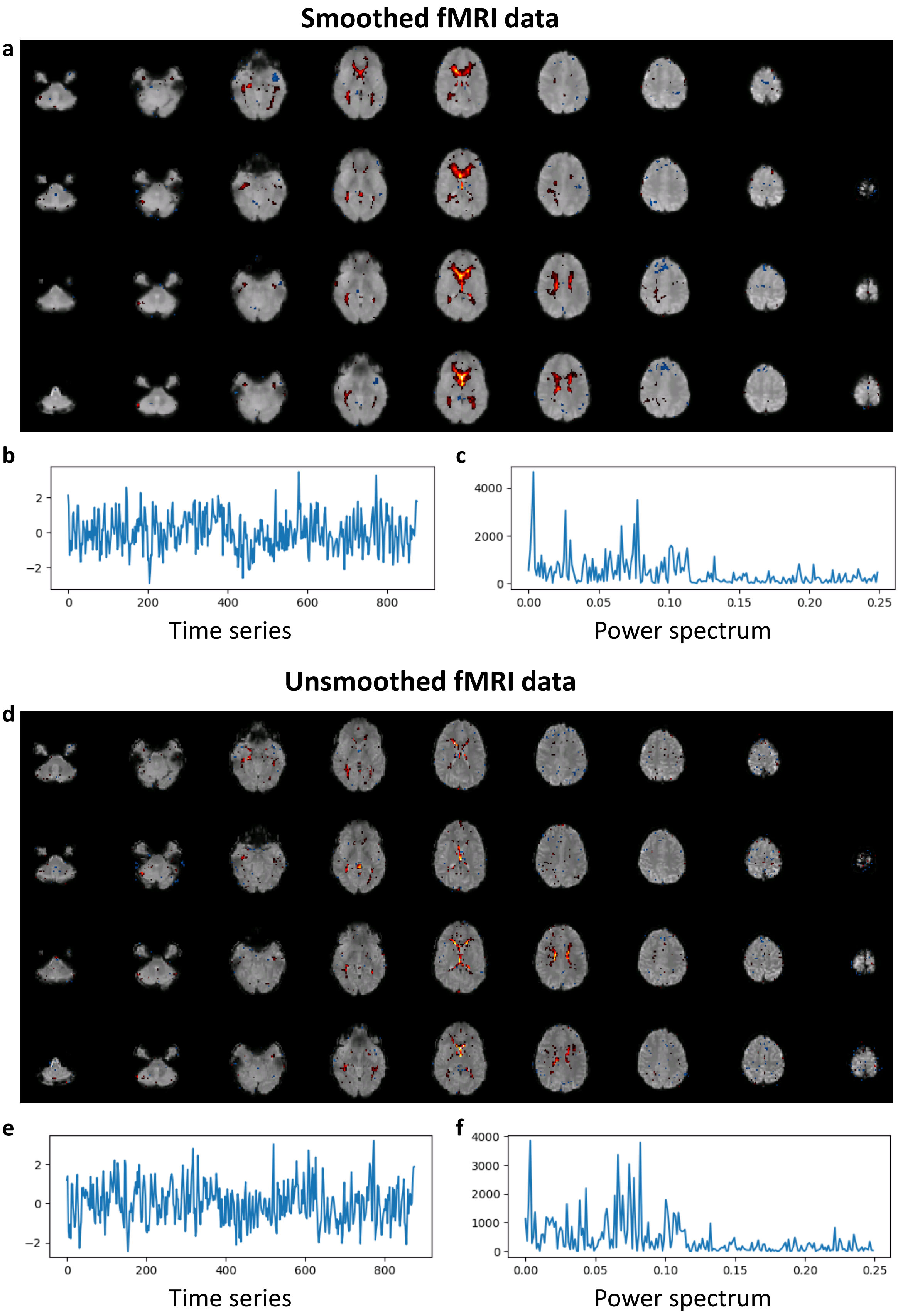

Supplementary Figure 4. Example ICs classified as CSF artifacts (top: smoothed fMRI data [sub-03, run-3, IC-082]; bottom: unsmoothed fMRI data [sub-03, run-3, IC-097]). The spatial map of the ICs (thresholded at Z > 2.3) showed that the voxels with large intensities mainly located in the CSF, especially the lateral ventricle **(a, d)**. The time series of the ICs showed a high-frequency oscillation pattern, without sudden jumps or gradual change **(b, e)**. A large portion of the power of the ICs' time series appeared beyond the low-frequency band (i.e., 0.01-0.1Hz) **(c, f)**.

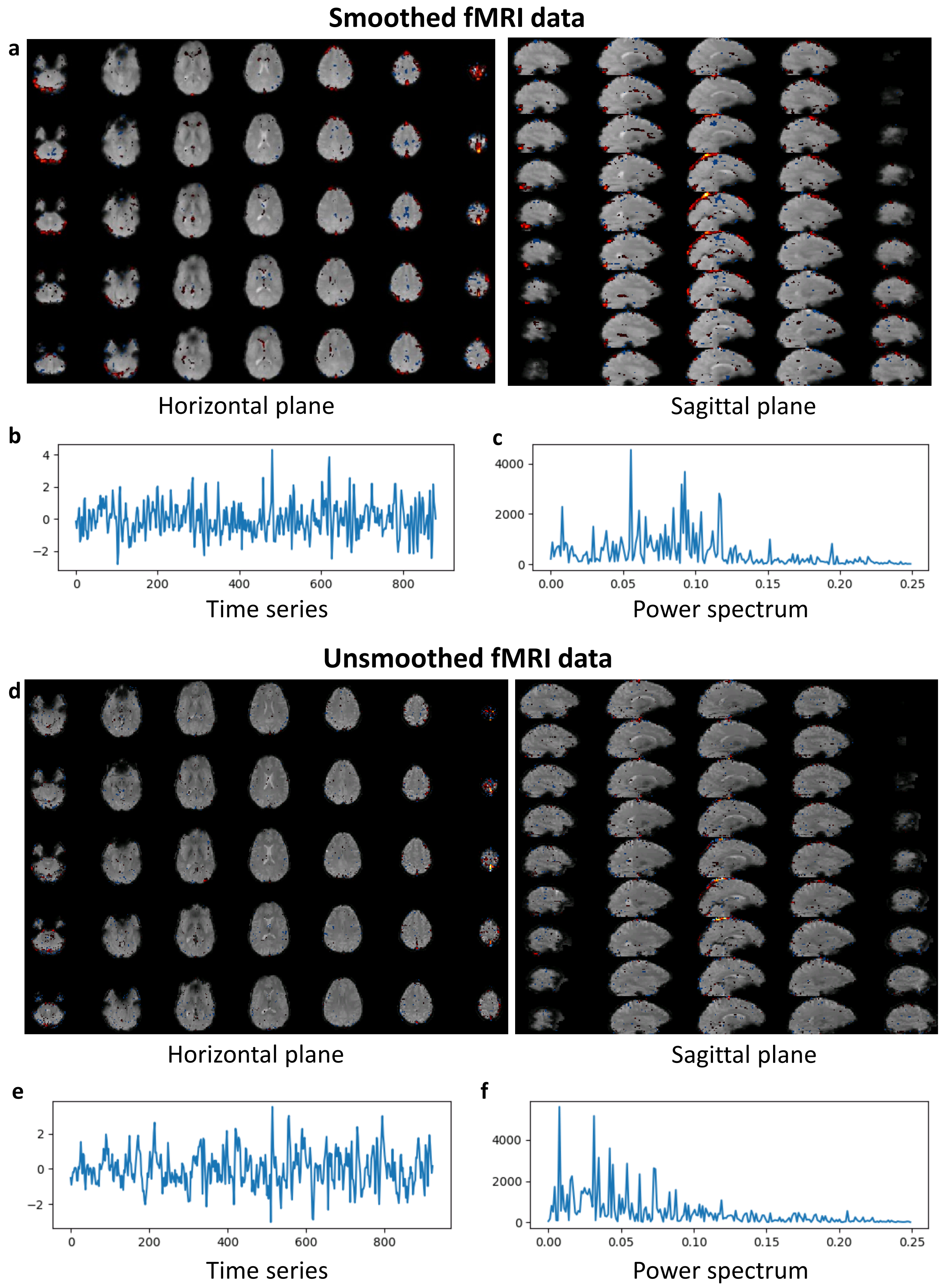

Supplementary Figure 5. Example ICs classified as veins artifacts (top: smoothed fMRI data [sub-01, run-2, IC-023]; bottom: unsmoothed fMRI data [sub-01, run-5, IC-055]). The spatial map of the ICs (thresholded at $Z > 2.3$) showed that the voxels with large intensities mainly located in veins, mostly the sagittal sinus, which could be observed more clearly from the sagittal view **(a, d)**. The time series of the ICs showed a low-frequency oscillation pattern **(b, e)**. A large portion of the power of the ICs' time series appeared within the low-frequency band (i.e., 0.01-0.1Hz) **(c, f)**.

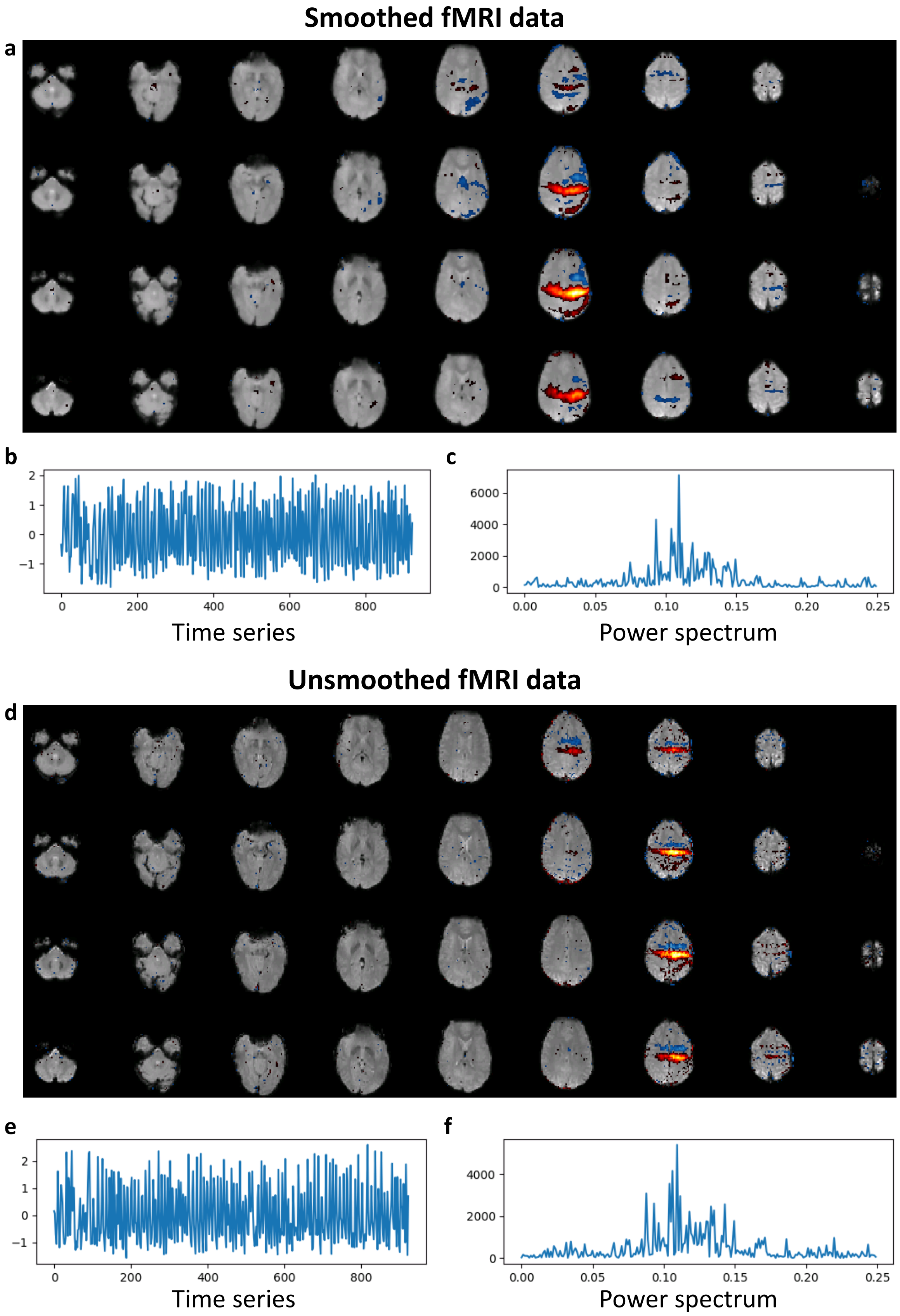

Supplementary Figure 6. Example ICs classified as white matter artifacts (top: smoothed fMRI data [sub-05, run-5, IC-008]; bottom: unsmoothed fMRI data [sub-05, run-5, IC-024]). The spatial map of the ICs (thresholded at $Z > 2.3$) showed that the voxels with large intensities mainly located in the white matter or deep veins **(a, d)**. The time series of the ICs showed a high-frequency oscillation pattern, without sudden jumps or gradual change **(b, e)**. A large portion of power of the ICs' time series appeared beyond the low frequency band (i.e., 0.01-0.1Hz) **(c, f)**.

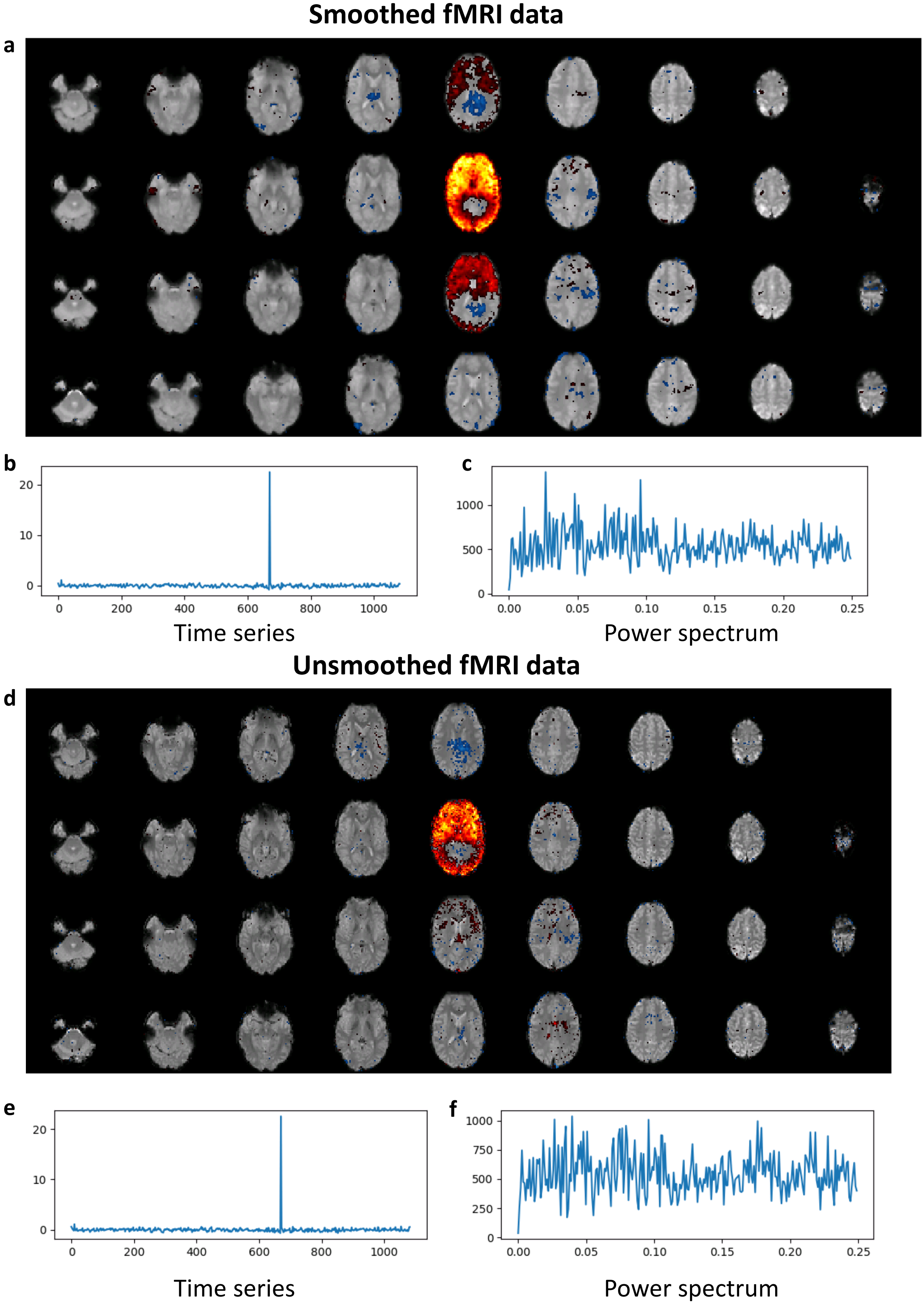

Supplementary Figure 7. Example ICs classified as MRI-related artifacts (top: smoothed fMRI data [sub-06, run-7, IC-001]; bottom: unsmoothed fMRI data [sub-06, run-7, IC-001]). The spatial map of the ICs (thresholded at Z > 2.3) showed that the voxels with large intensities only appeared in one slice or several consecutive slices, and largely overlapped with non-grey matter area **(a, d)**. The time series of the ICs showed a clear spike **(b, e)**. The spectral power of the ICs' time series distributed uniformly **(c, f)**.

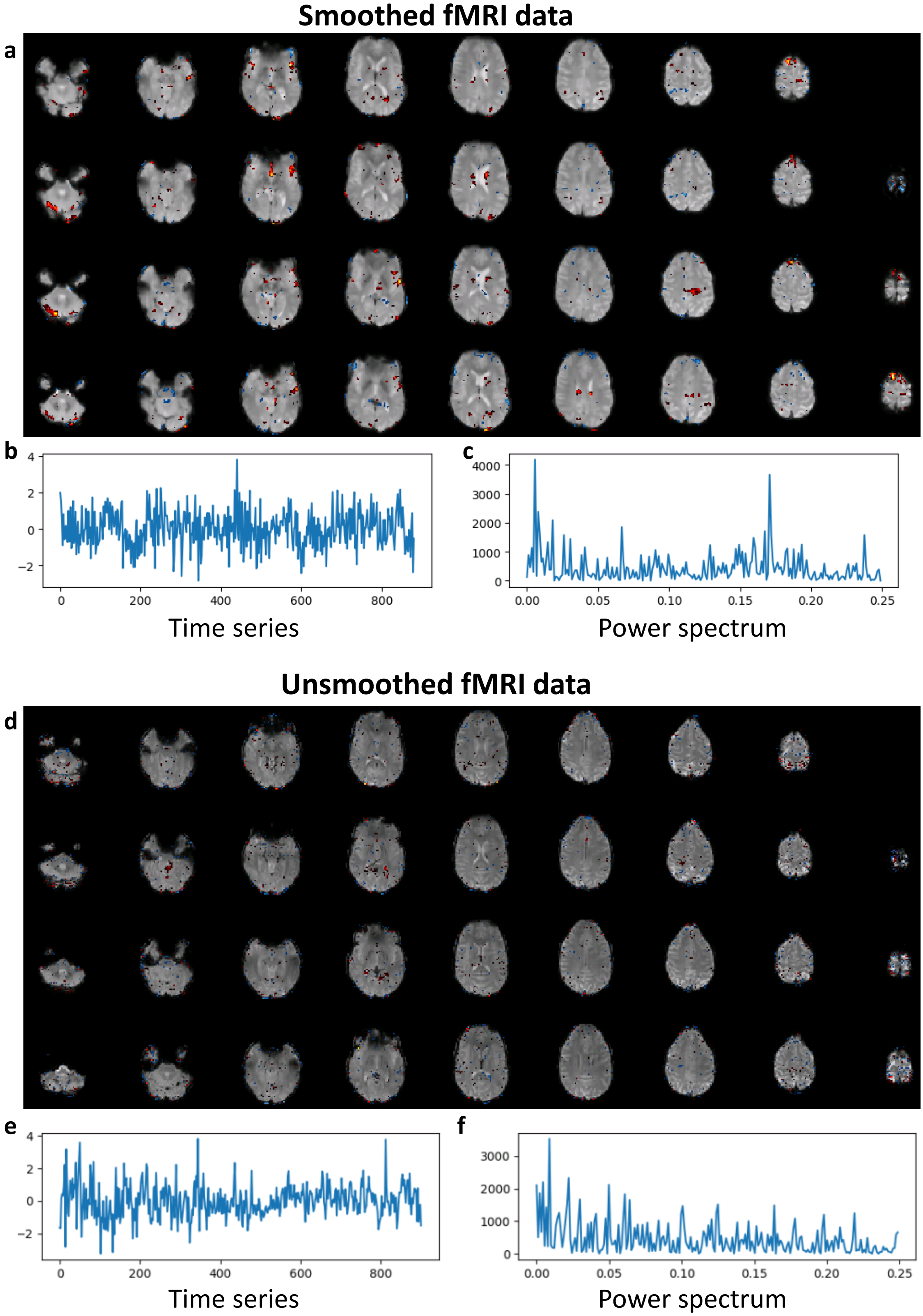

Supplementary Figure 8. Example ICs classified as unclassified noise (top: smoothed fMRI data [sub-02, run-2, IC-092]; bottom: unsmoothed fMRI data [sub-19, run-1, IC-110]). The ICs might contain several sources of noise. The spatial map of the ICs (thresholded at Z > 2.3) showed that the voxels with large intensities sparsely distributed in several non-grey matter regions corresponding to different source of artifacts, like in the white matter, arteries, veins, or out of the brain **(a, d)**. The time series of the ICs showed mixed characteristics of different artifacts **(b, e)**. Again, the spectral power the ICs' time series revealed that both high-frequency and low-frequency fluctuations were mixed together **(c, f)**.

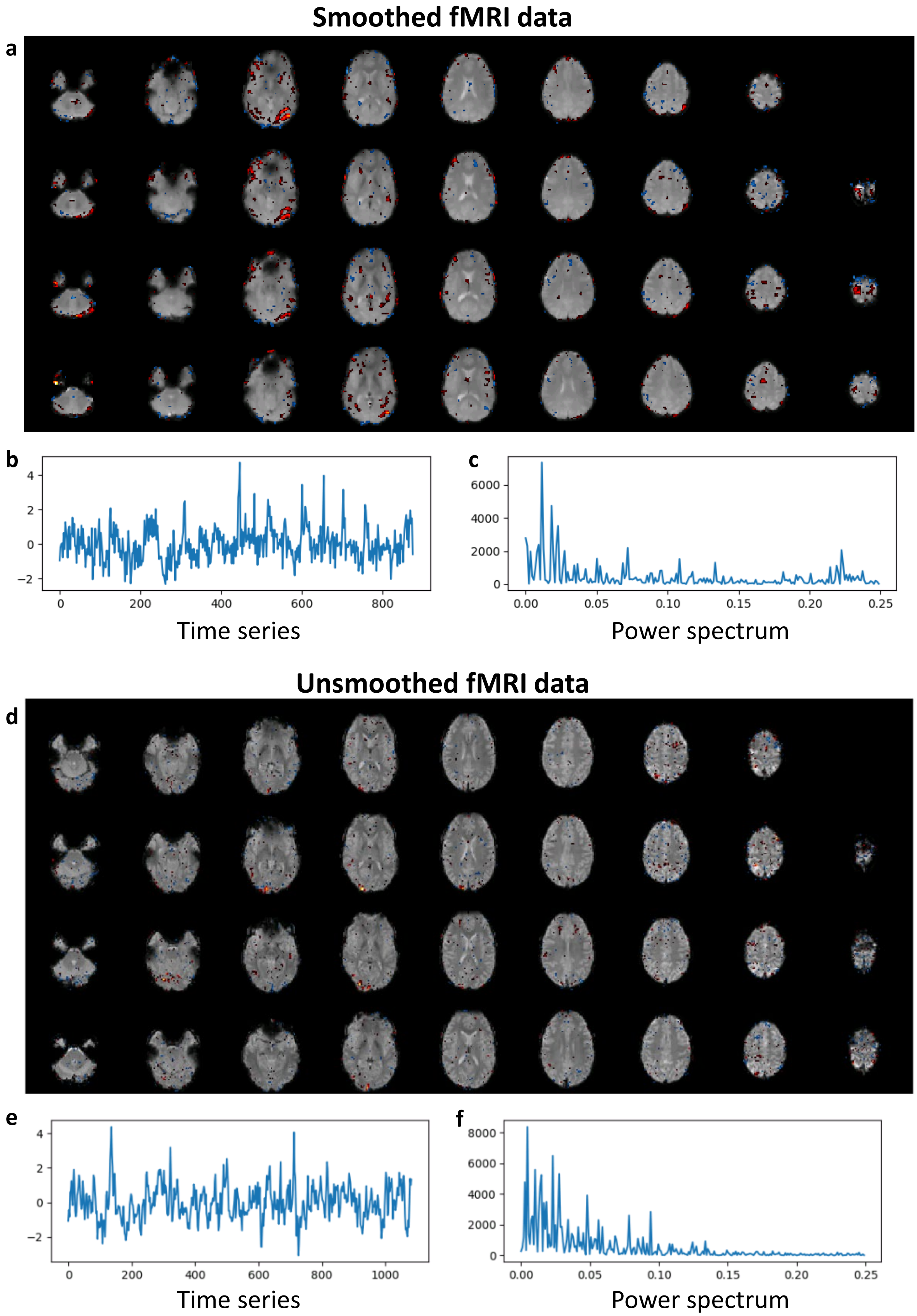

Supplementary Figure 9. Example ICs classified as unknown signal (top: smoothed fMRI data [sub-01, run-3, IC-086]; bottom: unsmoothed fMRI data [sub-06, run-7, IC-165]). The spatial map of the ICs (thresholded at $Z > 2.3$) showed that the voxels with large intensities appeared only partially in the grey matter **(a, d)**. The time series of the ICs showed a low-frequency oscillation pattern, with possible sudden jumps due to head motion or other artifacts **(b, e)**. The spectral power of the ICs' time series lied primarily in low-frequency band (i.e., 0.01-0.1Hz) **(c, f)**.

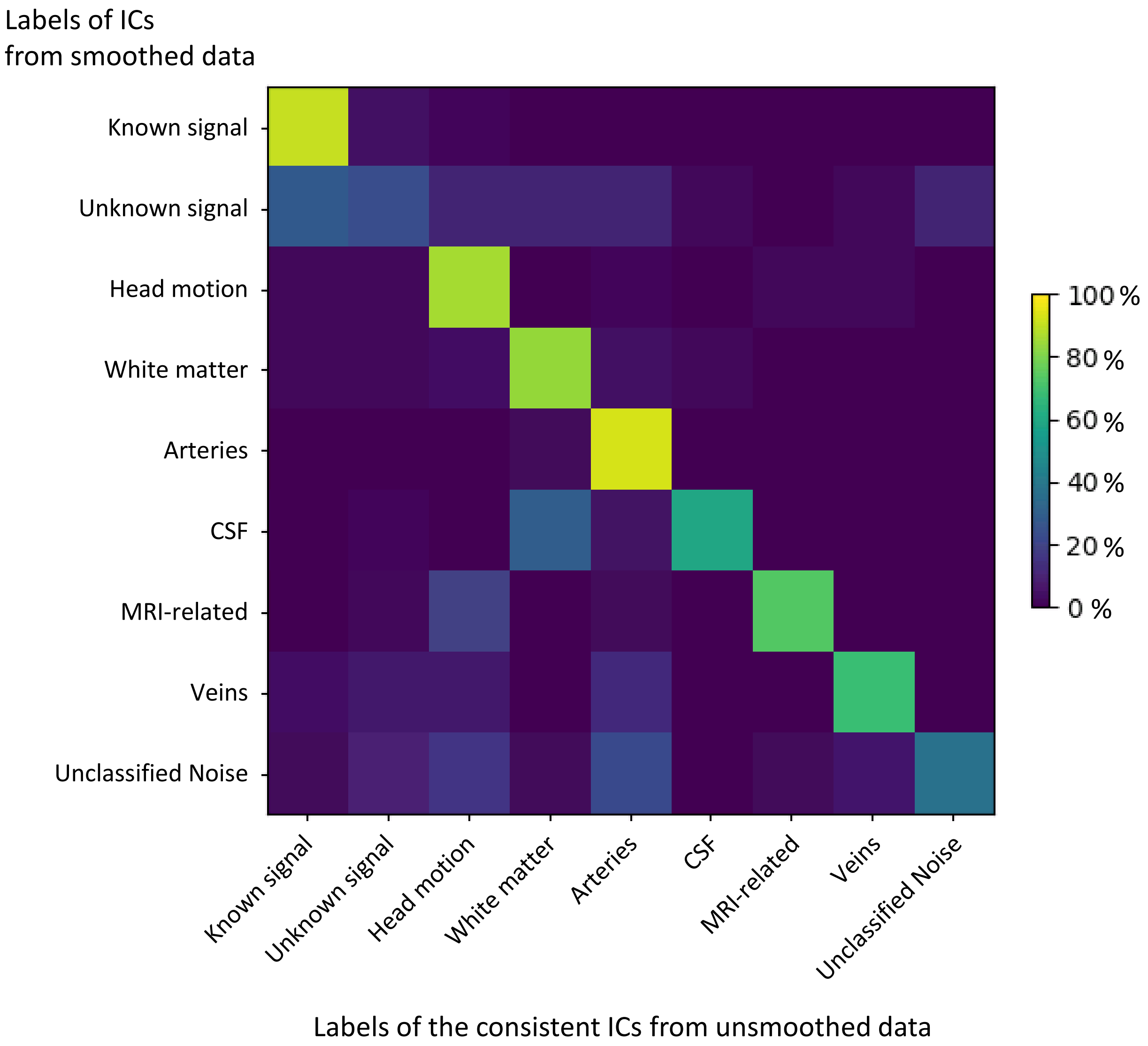

Supplementary Figure 10. The confusion matrix of IC classifications for consistent IC pairs from the smoothed data and the unsmoothed data. In the confusion matrix, the cell located in row i and column j indicated the percentage of ICs classified as the *i*th category whose consistent IC from the corresponding unsmoothed data was classified as the *j*th IC category. The figure revealed that the consistent IC pairs were generally classified into the same category out of nine categories (on average = 82.34%) despite of some mismatches. The mismatches mainly happened in the classification of unknown signal and unclassified noise.

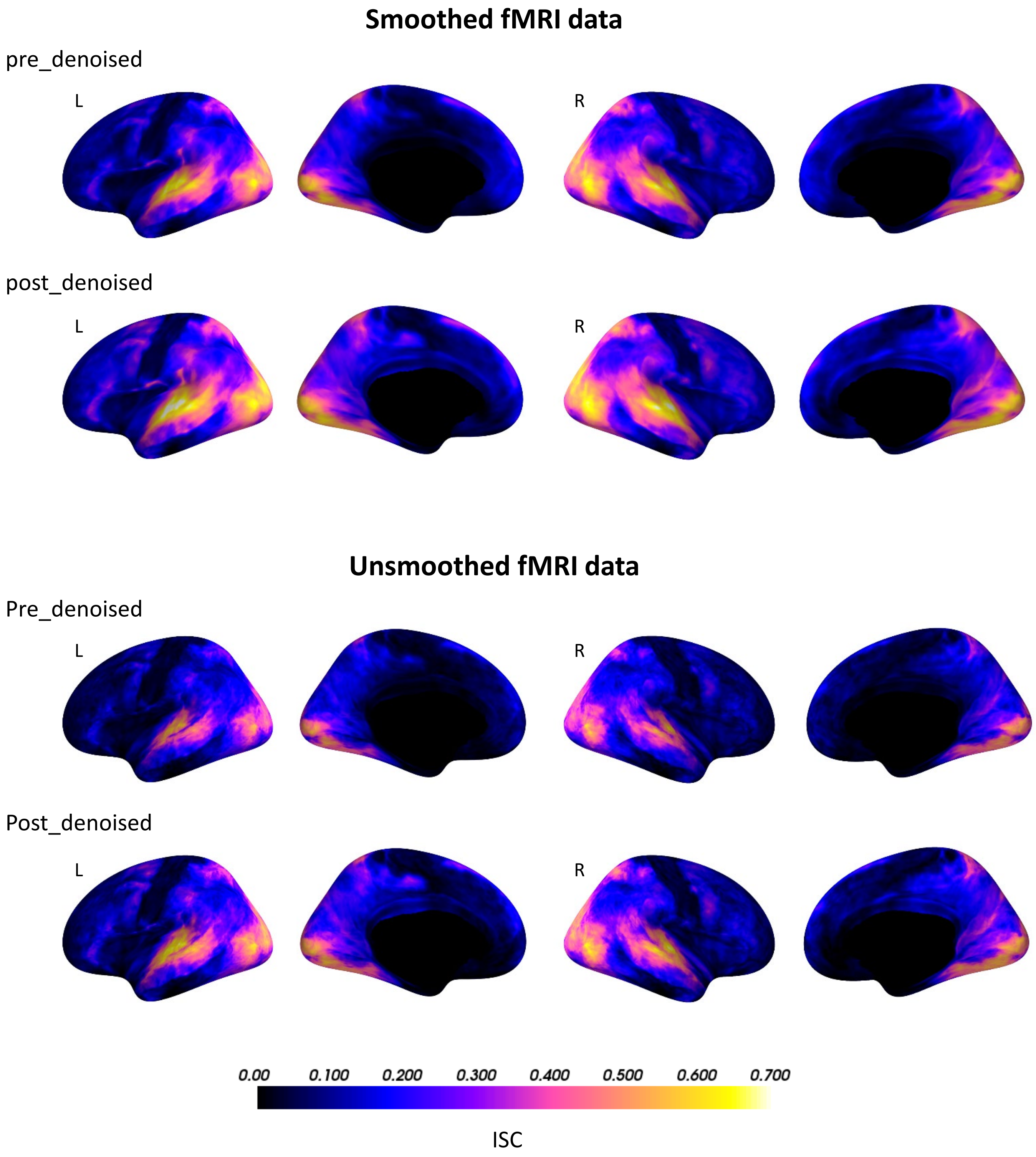

Supplementary Figure 11. The ISC calculated from the pre- and post-denoised data for each vertex on the fsaverage surface (top: smoothed fMRI; bottom: unsmoothed fMRI data). Consistent with previous studies on ISC during movie watching, visual cortices, auditory cortices, precuneus, superior temporal sulcus and temporal parietal junction showed high ISC in both the pre- and post-denoised data. On the other hand, several areas showed low ISC (close to zero) in both the pre- and post-denoised data including primary motor cortex, somatosensory cortex and medial prefrontal cortex. The denosing procedure generally increased the ISC for areas with high ISC whereas decreased the ISC for areas with low ISC (Figure 4). The disassociation of the denoising effects in different areas indicated that the our denoising procedure specifically enhanced neural-related ISC and weakened ISC from non-neural sources.
